# Association between TMD signs and symptoms, condyle alterations and joint effusion: an MRI retrospective study

**DOI:** 10.1590/1807-3107bor-2026.vol40.070

**Published:** 2026-08-24

**Authors:** Jhonatan Thiago Lacerda-Santos, Gélica Lima Granja, Gabriela Dias Prado, Janaina Araújo Dantas, Paulo Sérgio Flores Campos, Patrícia Meira Bento, Daniela Pita de Melo

**Affiliations:** 1 Universidade Estadual da Paraíba – UEPB, Department of Oral Diagnosis, Division of Oral Radiology, Campina Grande, PB, Brazil.; 2 Universidade Estadual da Paraíba – UEPB, Department of Dentistry, Campina Grande, PB, Brazil.; 3 Universidade Federal da Bahia – UFBA, School of Dentistry, Department of Oral Radiology, Salvador, BA, Brazil.; 4 Universidade Federal de Sergipe – UFS, School of Dentistry, Department of Dentistry, Aracaju, SE, Brazil.; 5 University of Saskatchewan, College of Dentistry, Saskatoon, Canada.

**Keywords:** Temporomandibular Joint, Temporomandibular Joint Disorders, Magnetic Resonance Imaging

## Abstract

This study aimed to assess whether signs, symptoms, and condylar changes are associated with temporomandibular joint (TMJ) effusion. An observational, cross-sectional, retrospective study was carried out on 1,182 TMJ magnetic resonance imaging (MRI) scans from 591 patients. Images of patients who underwent MRIs of the temporomandibular joints were included. The MRIs were obtained from the records of a private radiology clinic and were acquired in 2018. Two calibrated observers assessed effusion and condylar changes. The evaluated signs and symptoms were joint pain, crepitation, clicking, irregular mouth opening movement, and limited mouth opening. Descriptive statistics were performed, followed by unadjusted and adjusted binary logistic regression analyses to calculate the odds ratio (OR) and 95% confidence interval (95%CI), with a significance level of p ≤ 0.05. The analysis of the variables was performed using the individual as the unit of grouping, with clustered robust standard errors (using the Huber-White estimator) adjusted at the patient level. The prevalence of effusion was over 51% in the studied sample (51.6% right TMJ; 52.8% left TMJ). The most prevalent condylar alterations were condylar erosion and limited condyle width. In the final model, patients aged 50 to 59 years (OR: 2.54; 95%CI: 1.17–5.50), those with clicking (OR: 1.59; 95%CI: 1.07–2.37) and those with condylar erosion (OR: 2.84; 95%CI: 1.56–5.28) were more likely to have joint effusion. It is concluded that joint effusion has a high prevalence in individuals aged 50–59 years, followed by those aged 20 to 29 years, and may be associated with the presence of clicking and condylar erosion.

## Introduction

The temporomandibular joint (TMJ) is a complex ginglymoarthrodial joint because it allows both hinge or rotational (ginglymoid) movements and sliding or translational (arthrodial) movements. It is the only joint in the human body capable of performing such complex and diverse movements.^1-3^ The TMJ can be affected by disorders that compromise psychosocial and physical functioning, negatively impacting an individual's quality of life^4,5^.

The American Academy of Orofacial Pain (AAOP) defines temporomandibular disorders (TMD) as a set of conditions that affect the muscles of mastication, the bones and ligaments of the temporomandibular joint (TMJ), and related structures.^6,7^ Patients with TMD often experience pain, clicking, or crackling during mandibular movements, as well as limited mouth opening.^2,8^

TMD has a multifactorial etiology that involves biopsychosocial factors such as stress, anxiety, and depression, along with local factors including trauma, malocclusion, and parafunctional habits.^9,10^ TMJ functional overload can cause biochemical changes within the joint that lead to oxidative stress and the production of free radicals, which modify the composition and volume of joint fluid.^2^ Excessive fluid accumulation in the joint space due to an inflammatory reaction leads to joint effusion and can be assessed through magnetic resonance imaging (MRI).^11,12^

MRI is a noninvasive, nonionizing examination that offers excellent precision, reliability, and resolution. It is considered the gold standard exam for the visualization of soft tissues, internal fluids, and morphological and inflammatory changes in the TMJ.^7,13-16^ On MRI, effusion is characterized by high signal intensity on T2-weighted spin-echo sequences.^16,17^

Effusion is highly prevalent, ranging from 10.1% to 68.10%.^12,18^ It is primarily caused by inflammation of the synovial membrane, which leads to increased fluid production and results in joint swelling, pain, and stiffness. Joint effusion can result from trauma, injury, or degenerative changes in the joint and is considered an indicator of an inflammatory process.^19,20^

Previous studies have examined the relationship between effusion, pain, limited mouth opening, and anterior disc displacement without reduction.^8,11,12,15,21^ However, they have not evaluated condylar changes associated with joint effusion. If a link is found between condylar alterations and joint effusion, it could provide important diagnostic information and aid in the early diagnosis of effusion.

Studies investigating the clinical and radiographic aspects associated with effusion are fundamental for TMD diagnosis and treatment planning. Therefore, this study aimed to assess whether signs, symptoms, and condylar changes are associated with TMJ effusion.

## Methods

This research was approved by the local Human Research Ethics Committee (n: 00683418.2.0000.5024) and developed in accordance with the ethical precepts of the Declaration of Helsinki. An observational, cross-sectional, retrospective study was carried out on 591 patients, 591 MRI examinations, and 1,182 TMJs. All patients signed an informed consent form. Images of patients who underwent MRI of the temporomandibular joints were included. Patients with rheumatic diseases, a history of TMJ surgery, orthognathic surgery, TMJ trauma, or facial bone fractures were excluded from the sample.

The sample was selected by convenience. The MRI scans were obtained from the collection of a private radiology clinic, acquired between January 2018 and December 2019. The exams were performed using a Signa 1.5T MR system (General Electric Medical Systems, Milwaukee, USA) with a bilateral surface coil. First, parasagittal and coronal proton density-weighted images were acquired in both closed-mouth and open-mouth positions. Then, parasagittal T2-weighted images with fat saturation were acquired in the closed-mouth position. All images were obtained in optimal coronal, sagittal, and axial planes relative to the condyle inclination. The images were stored in a PACS (Picture Archiving and Communication System) (Carestream PACS suite, version 11.4.1.1011, Carestream Health, Rochester, USA).

The images were analyzed by an oral and maxillofacial radiologist with more than 25 years of experience, considered the gold standard, who issued the effusion report. A second oral and maxillofacial radiologist with 7 years of experience interpreting TMJ images was trained and calibrated by the standard examiner using 20 MRI images (inter-examiner kappa coefficient = 0.823). For intra-examiner calibration, 30% of the MRI image sample was assessed twice, with a 60-day interval between assessments, resulting in high intra-examiner agreement (intra-examiner kappa coefficient = 0.968) for the diagnosis of condylar alterations.

The training for clinical data collection was based on the Diagnostic Criteria for TMDs (DC/TMD) Axis I.^4^ During calibration, a trained examiner evaluated 20 patients for TMD signs and symptoms, joint noise (crepitation and clicking), and mouth opening. The intra-examiner kappa coefficients for joint noise (k = 0.927) and mouth opening (k = 0.951) were substantial.

**Figure 1:**
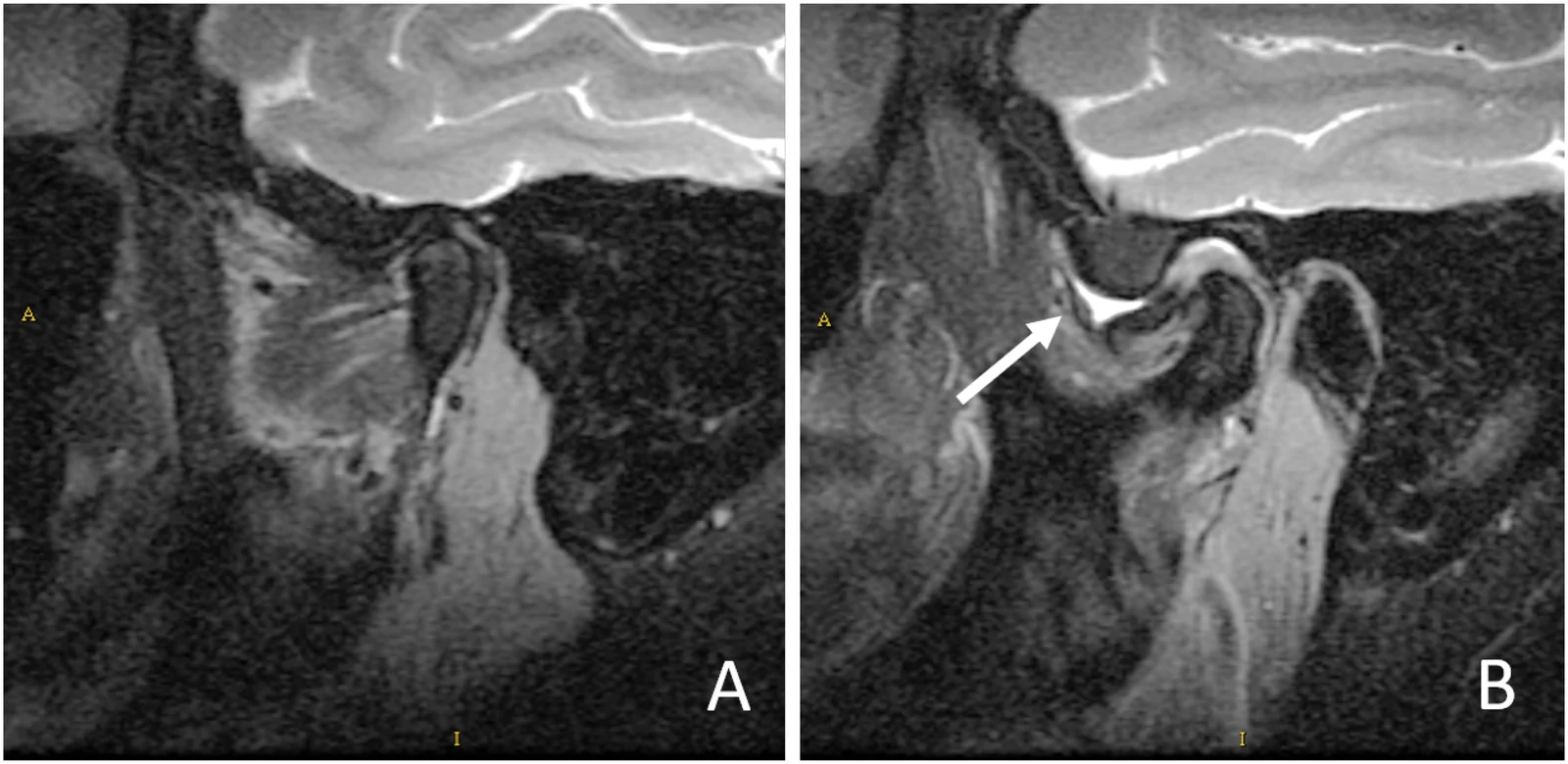
MRI sagittal images of the TMJ:. A) without effusion; B) with effusion.

**Figure 2:**
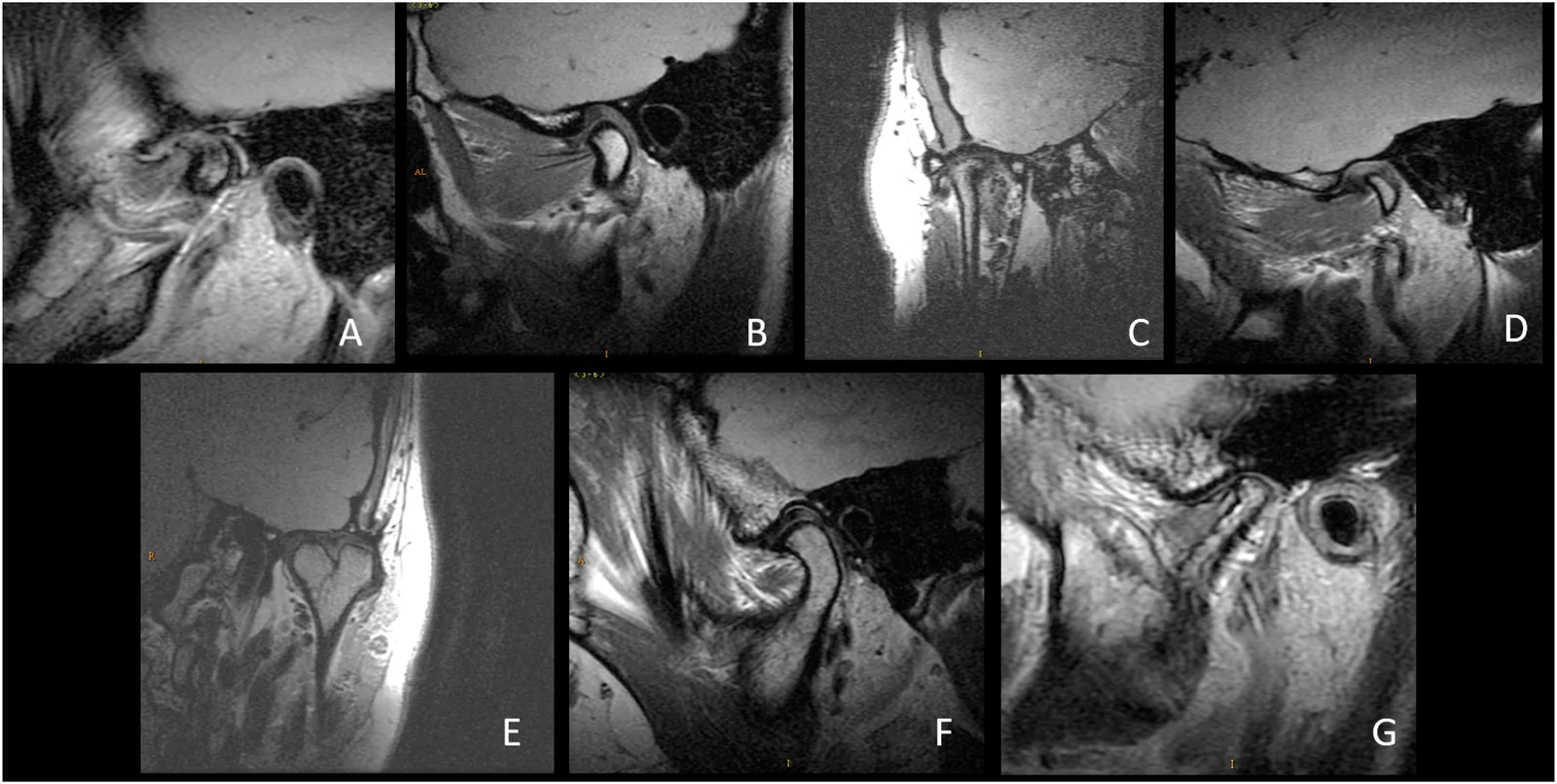
MRI images of the TMJ showing examples of the assessed condylar alterations: A - erosion; B - condylar cortical thickening; C - limited condyle width; D - limited condyle thickness; E - presence of bifid condyle/rudimentary bifidity; F - condylar hyperplasia; G - condylar hypoplasia.

In the present study, effusion was categorized as either present or absent (Figure 1). Additionally, the following condylar changes were assessed: erosion, condylar cortical thickening, limited condyle width, limited condyle thickness, presence of bifid condyle/rudimentary bifidity or condylar depression, and condylar hypoplasia as well as hyperplasia (Figure 2). Hypoplasia was defined as a condyle thickness of less than 5 mm and a mediolateral width of less than 1.5 cm. Hyperplasia was diagnosed when condyle thickness exceeded 5 mm and mediolateral width exceeded 1.5 cm.

The assessed signs and symptoms included joint pain, crepitation, clicking, irregular mouth opening movement, and limited mouth opening. Before the MRI scan, an oral and maxillofacial radiologist (PSFC), trained in TMJ evaluation with 25 years of experience, conducted a clinical examination of all subjects according to the DC/TMD Axis I.^4^ During the examination, the presence of joint noises (clicking or crepitation) audible during jaw movements was observed by auscultation with a stethoscope. Mouth opening was evaluated by measuring the distance between the maxillary and mandibular incisors using a digital caliper, and aperture limitation was considered present when the distance was less than 40 mm^4^. The VAS scale was used to evaluate pain intensity based on self-report during the clinical examination. According to the instrument, pain levels ranged from 0 (no pain) to 10 (unbearable pain). Additionally, the sex of the subjects and the presence of joint pain, as reported by the subjects, were recorded.

To perform the statistical analysis, participants’ ages were grouped according to the methodology described by Jeon et al.^8^ As the objective of this study was to estimate the clinical and condylar factors associated with the presence of effusion, this variable was dichotomized as present or absent.^21-23^ Unadjusted and adjusted binary logistic regression analyses were performed to calculate the odds ratio (OR) and 95% confidence interval (95%CI), using joint effusion as the dependent variable. A significance level of p ≤ 0.05 was adopted. Variables with p < 0.20 in the unadjusted analysis were included in the adjusted model. To account for possible correlation between joints within the same individual, the analysis was performed using the individual as the grouping unit, with clustered robust standard errors (Huber-White estimator) adjusted at the patient level. Variables that provided a better fit through backward elimination and were adjusted using the Hosmer-Lemeshow test remained in the final model: age group, clicking, and condylar erosion. The sociodemographic variables used as controls included sex and age.

## Results

The distribution of variables is detailed in Table 1. Of the assessed patients, 95.8% reported signs and symptoms, particularly pain (69.5%) and clicking (69.7%) The prevalence of effusion was over 51% in the studied sample (51.6% right TMJ; 52.8% left TMJ). The most prevalent condylar alterations were condylar erosion (12.5% right TMJ; 14.6% left TMJ), and limited condyle width (11.0% right TMJ; 10.8% left TMJ) (Table 2).

**Table 1:** Frequency of sex, age, joint signs and symptoms variables (n = 591).

Variables		n (%)
Sex		
	Male	115 (19.5)
	Female	476 (80.5)
Age (years)		
	9–19	50 (8.5)
	20–29	147 (24.9)
	30–39	174 (29.4)
	40–49	93 (15.7)
	50–59	61 (10.3)
	60–69	46 (7.8)
	≤ 70	20 (3.4)
TMD signs and symptoms		
	Absent	25 (4.2)
	Present	566 (95.8)
Pain		
	Absent	168 (30.5)
	Present	411 (69.5)
Crepitation		
	Absent	433 (73.3)
	Present	158 (26.7)
Clicking		
	Absent	179 (30.3)
	Present	412 (69.7)
Irregular mouth opening movement		
	Absent	589 (99.7)
	Present	02 (0.3)
Limited mouth opening		
	Absent	378 (64.0)
	Present	213 (36.0)

**Table 2:** Frequency of joint effusion and condylar alterations (n = 1.182).

Variables		Right TMJ	Left TMJ
		n (%)	n (%)
Effusion			
	Absent	286 (48.4)	279 (47.2)
	Present	305 (51.6)	312 (52.8)
Condylar erosion			
	Absent	517 (87.5)	505 (85.4)
	Present	74 (12.5)	86 (14.6)
Condylar cortical thickening			
	Absent	586 (99.2)	586 (99.2)
	Present	05 (0.8)	05 (0.8)
Bifid condyle, rudimentary bifidity, or condylar depression			
	Absent	589 (99.7)	588 (99.5)
	Present	02 (0.3)	03 (0.5)
Limited condyle width			
	Absent	526 (89.0)	527 (89.2)
	Present	65 (11.0)	64 (10.8)
Limited condyle thickness			
	Absent	508 (86.0)	519 (87.8)
	On its full extension	30 (5.1)	26 (4.4)
	On its lateral portion	42 (7.1)	34 (5.8)
	On its medial portion	08 (1.4)	09 (1.5)
	On its central portion	03 (0.5)	03 (0.5)
Condylar hypoplasia			
	Absent	553 (93.6)	548 (92.7)
	Present	38 (6.4)	43 (7.3)
Condylar hyperplasia			
	Absent	588 (99.5)	591 (100.0)
	Present	03 (0.5)	0 (0.0)

The variables pain, crepitation, limitation of mouth opening, and irregular movement of mouth opening were not associated with the presence of joint effusion. In the final model, the variables age 50 to 59 years (OR: 2.54; 95%CI: 1.17–5.50), clicking (OR: 1.59; 95%CI: 1.07–2.37), and condylar erosion (OR: 2.84; 95%CI: 1.56–5.28) remained associated with the presence of effusion (Table 3).

**Table 3:** Binary logistic regression in relation to joint effusion associated with sex, age, presence of clinical signs and symptoms, and condylar changes.

Variable		Joit effusion		Bivariate		Multivariate	
		Absent	Present	Non-adjusted OR**		Adjusted OR**	
		n (%)	n (%)	p-value	(95%CI)	p-value	(95%CI)
Sex (591)							
	Male	57 (49.6)	58 (50.4)		1		–
	Female	229 (48.1)	247 (51.9)	0.77	1.06 (0.70–1.59)	–	–
Age (years) (591)							
	9–19	30 (60.0)	20 (40.0)		1		1
	20–29	58 (39.5)	89 (60.5)	0.01	2.30 (1.19–4.43)	0.01	2.27 (1.18–4.38)
	30–39	95 (54.6)	79 (45.4)	0.49	1.24 (0.65–2.36)	0.47	1.26 (0.66–2.38)
	40–49	50 (53.8)	43 (46.2)	0.47	1.29 (0.64–2.59)	0.49	1.27 (0.63–2.55)
	50–59	22 (36.1)	39 (63.9)	0.01	2.65 (1.23–4.74)	0.01	2.54 (1.17–5.50)
	60–69	22 (47.8)	24 (52.2)	0.23	1.63 (0.72–3.67)	0.39	1.42 (0.62–3.21)
	≥ 70	9 (45.0)	11 (55.0)	0.25	1.83 (0.64–5.22)	0.35	1.71 (0.54–5.38)
TMD signs and symptoms (1,182)							
	Absent	17 (68.0)	8 (32.0)		1		–
	Present	269 (47.5)	297 (52.5)	0.05	2.34 (0.99–5.52)	–	–
Pain (591)							
	Absent	85 (47.2)	95 (52.8)		1		
	Present	201 (48.9)	210 (51.1)	0.70	1.07 (0.75–1.51)	–	–
Crepitation (591)							
	Absent	221 (51.0)	212 (49.0)		1		
	Present	65 (41.1)	93 (58.9)	0.03	1.49 (1.03–2.15)	–	–
Clicking (591)							
	Absent	107 (59.8)	72 (40.2)		1		1
	Present	179 (43.4)	233 (56.6)	< 0.001	1.93 (1.35–2.76)	0.02	1.59 (1.07–2.37)
Irregular mouth opening movement (1,182)							
	Absent	286 (48.6)	303 (51.4)		–	–	–
	Present	0 (0.0)	2 (100.0)	0.50	–	–	–
Limited mouth opening (1,182)							
	Absent	190 (50.3)	188 (49.7)		1		–
	Present	96 (45.1)	117 (54.9)	0.22	1.23 (0.87–1.72)	–	–
Condylar erosion (1,182)							
	Absent	520 (50.9)	502 (49.1)		1		1
	Present	45 (28.1)	115 (71.9)	< 0.001	2.64 (1.83–3.81)	0.001	2.84 (1.56–5.28)
Bifid condyle, rudimentary bifidity, or condylar depression (1,182)							
	Absent	564 (47.9)	613 (52.1)		1		–
	Present	1 (20.0)	4 (80.0)	0.24	3.68 (0.41–33.02)	–	–
Condylar cortical thickening [1.182]							
	Absent	563 (48.0)	609 (52.0)		1		–
	Present	2 (20.0)	8 (80.0)	0.09	3.69 (0.78–17.48)	–	–
Limited condyle width [1.182]							
	Absent	506 (48.1)	547 (51.9)		1		–
	Present	59 (45.7)	70 (54.3)	0.61	1.09 (0.76–1.58)	–	–
Limited condyle thickness [1.182]							
	Absent	495 (48.2)	532 (51.8)		1		–
	Present	70 (45.2)	85 (54.8)	0.48	1.13 (0.80–1.56)	–	–
Condylar hypoplasia [1.182]							
	Absent	532 (48.3)	569 (51.7)		1		–
	Present	33 (40.7)	48 (59.3)	0.18	1.36 (0.86–2.15)	–	–
Condylar Hyperplasia [1.182]							
	Absent	564 (47.8)	615 (52.2)		1		–
	Present	1 (33.3)	2 (66.7)	0.62	1.83 (0.16–20.28)	–	–

** Unadjusted binary logistic regression for independent variables and joint effusion;

** Binary logistic regression adjusted for independent variables and joint effusion.

The odds of having a joint effusion were 2.54 times higher in individuals aged 50–59 years and 2.27 times higher in those aged 20–29 years compared with other age groups studied. Patients with clicking were 1.59 times more likely to have a joint effusion than those without clicking. The odds of joint effusion were 2.84 times higher in patients with condylar erosion than in those without.

## Discussion

The results of this study showed a high prevalence of joint effusion in the 50–59-year age group, associated with the presence of clicking and condylar erosion, followed by the 20–29-year age group. Regarding the clinical findings, patients with joint clicking were found to be 59% more likely to have joint effusion. Joint clicking is characterized by a short-duration sound identified through auscultation during the clinical examination^6^. This association may arise because inflammatory cytokines in the retrodiscal tissue promote joint destruction and trigger arthritis, which is thought to produce the popping sound.^8^

The etiology of TMJ effusion remains unclear; however, it appears to be associated with a dysfunctional relationship between the mandibular condyle and disc. This dysfunction leads to the deterioration and abrasion of articular cartilage and disc surfaces, releasing pro-inflammatory cytokines that contribute to additional joint damage and the development of osteoarthritis.^24-27^

Pain is one of the main symptoms of TMD and can impact daily activities, psychosocial functioning, and the quality of life for patients affected by this condition.^4^ Previous studies have reported an association between pain and joint effusion;^8,15,27,28^ however small accumulations of fluid can also be observed in asymptomatic patients.^29,30^

The present study did not find an association between effusion and pain, which corroborates previous studies.^11,12,31,32^ However, Jeon et al.^8^ found that a moderate to severe degree of effusion is associated with pain. A possible explanation for these divergences may relate to the degree of effusion; as fluid accumulation increases, so does the intra-articular pressure, which can intensify pain symptoms. Additionally, pain perception is subjective and can be influenced by stress, anxiety, or it may represent a symptom of another disease in the upper neck region.^33^ Previous studies have suggested a link between anxiety and depression symptoms and TMD, as well as pain perception, which can hinder the effectiveness of TMD treatment. Effusion occurs independently of psychosocial variables as part of the inflammatory process associated with TMD progression. Therefore, anxiety, depression, hypervigilance to pain, sleep quality, and other psychosomatic characteristics can be considered confounding variables in pain perception that will be present in any TMD assessment.^34,35^

However, effusion is part of the inflammatory process that causes painful symptoms; therefore, the patient may feel pain even if the volume of joint effusion is small.^36^ Although pain is the main complaint when assessing TMD patients, the results found in this study can guide professionals in evaluating other clinical factors of the TMJ that may aid in the diagnosis of TMD. Thus, considering additional signs can assist in the early diagnosis of degenerative changes in the TMJ.

When analyzing the prevalence of effusion by age group, a significant association was noted among patients aged 20–29 and 50–59. However, those in their fifth decade of life were 2.54 times more likely to experience joint effusion. Older patients likely did not seek early TMD treatment, leading to fluid accumulation in the area. On the other hand, another finding of this study shows that young patients (in their second decade of life) also had a high probability of effusion. This generation may have greater access to health information, prompting them to seek treatment for reported symptoms. Therefore, the detection of effusion by MRI may be linked to both early pursuit of treatment and advancing age.

Joint effusion and synovitis are common manifestations of rheumatic disease, playing an important role in disease pathophysiology. Early detection and accurate assessment can facilitate appropriate clinical management and improve patient prognosis.^37^ Effusions are also associated with abnormal disc position and a greater likelihood of anterior disc displacement without reduction, which can result in TMJ pain, restricted mouth opening, and changes in the patient's mouth opening trajectory.^38^

Previous studies have not identified an association between age and joint effusion, possibly due to differences in age group categorization, as seen in the study by Koca et al.,^15^ which used a wide age range of sample categorization. Another factor that may explain the absence of this association is the exclusion of the age variable from the regression analysis^27,32^. Given that the present study included 591 patients with 1,182 TMJ data points, a larger sample size than those of Koca et al.,^15^ Higuchi et al.,^27^ and Reverand et al.,^32^ the variations in sample sizes may have resulted in different outcomes.

Another finding of this study was the association between joint effusion and condylar erosion. Individuals with condylar erosion were found to be 2.92 times more likely to experience joint effusion. Condylar erosion is a significant bone change that indicates the absence or disruption of the cortical lining of the mandibular condyle.^27^ This association may be explained by the release of pro-inflammatory cytokines (tumor necrosis factor-alpha, interleukin-6, and interleukin-8) present in joint effusion, which is associated with joint degeneration.^39^ Therefore, effusion may indicate the onset of a more severe degenerative process if not diagnosed and treated promptly. This topic remains under discussion in the literature, and understanding the factors linked to TMD and the development of joint effusion can help achieve an accurate diagnosis and guide appropriate treatment strategies.

The TMJ is a bilateral joint that functions as a unit; therefore, when a disorder affects one side, it can also impact the other side, as the opposing TMJ attempts to compensate for the problem. This can lead to pain, dysfunction, and potentially the spread of symptoms. Longitudinal studies assessing both TMJs as a unit and observing the long-term effects of TMD and effusion on one side in relation to the opposite side can help clarify this issue.

This study has strengths, including its sample size, the use of a gold standard exam for diagnosing joint effusion, and the application of multivariate analysis. However, the main limitation of this study is its retrospective design, which does not allow for inferring causality. Although cross-sectional studies do not establish a cause-effect relationship, they aid in formulating hypotheses that guide longitudinal research and help prevent investigations from focusing on areas lacking significant clinical impact. Another limitation is that sociodemographic data were obtained from medical records, making it impossible to gather additional information such as patients’ income, marital status, and type of housing (urban or rural). Consequently, new prospective studies with larger sample sizes and more diverse populations are needed to analyze whether other demographic factors may influence the etiological factors of joint effusion and TMD. The etiology of joint effusion and its relationship with joint degeneration remain unclear; thus, longitudinal studies are required to determine whether joint effusion is an early marker of TMD or a consequence of the disorder's progression.

## Conclusion

The results indicate that joint effusion is highly prevalent in individuals aged 50–59, followed by those aged 20–29. Joint effusion may be associated with clicking and condylar erosion.

## Data Availability

The authors declare that all data generated or analyzed during this study are included in this published article.
